# Ecological determinants in plant community structure across dry afromontane forest patches of Northwestern Ethiopia

**DOI:** 10.1186/s12862-023-02176-0

**Published:** 2023-12-04

**Authors:** Metsehet Yinebeb, Ermias Lulekal, Tamrat Bekele

**Affiliations:** 1 Plant Biology and Biodiversity Management, College of Natural Sciences, Addis Ababa University, Addis Ababa, Ethiopia; 2 Biology Department, Kotebe University of Education, Addis Ababa, Ethiopia

**Keywords:** Species, Environmental factors, Plant communities, Gozamin, Forest patches

## Abstract

Ethiopia is a mountainous country with great geographic diversity. The diversified topographic features in Ethiopia made the country have a rich biodiversity forest cover in tropical Africa. This made Ethiopia have the largest floral diversity in tropical Africa. This floral diversity is rich in endemic elements. About 6,027 vascular plant species (including subspecies) with about 10.7% endemism have been documented. Plant community types are primarily influenced by topographic factors, as well as disturbance and environmental factors. The objective of this study is to demonstrate that 1: The forest patches in the study area exhibit distinct plant community types. 2: The composition and structure of these plant communities are influenced by various environmental variables. To achieve this, a total of 76 plots were used to collect vegetation and environmental data. The collected data were then analyzed using the R software, employing agglomerative hierarchical cluster analysis and redundancy analysis (RDA) to identify plant communities and assess the relationship between these communities and environmental variables.

R software was used to identify plant communities and analyze the relationship between plant community types and environmental variables using agglomerative hierarchical cluster analysis and redundancy analysis (RDA). Four plant community types were identified. The RDA results highlighted the significant impact (*p* < 0.005) of altitude, aspect, slope, grazing, and human interference on species distribution and the formation of plant communities. The RDA results highlighted the significant impact (*p* < 0.005) of altitude, aspect, slope, grazing, and human interference on species distribution and the formation of plant communities.

The findings indicate that the variation in plant communities is closely associated with topographic factors such as altitude, slope, aspect, as well as disturbance factors like grazing, and human interference, with altitude being the most influential factor. Based on these findings, it is recommended that conservation plans take into consideration the effects of grazing and human interference in order to address the challenges faced in conserving forest patches in the future.

Additionally, further research efforts should focus on mitigating disturbance factors and understanding the environmental variables that affect forest patches to enhance their conservation.

## Introduction

Ethiopia is a country with diverse environments and rich plant diversity [1]. Due to its unique biological characteristics, it serves as an important center of diversity and endemism, with a diverse flora [2]. The country’s flora is significantly diverse due to its climate variations, and it includes a substantial number of endemic species. It is estimated that Ethiopia has 6,027 vascular plants, out of which 10.7% are endemic [3]. The plant community refers to a group of plant species that grow together in a certain location and exhibit clear associations or affinities with one another [4]. Svenning et al. [5], state that the mechanisms responsible for shaping species-rich communities, such as those found in tropical rainforests, are a subject plant communities, are subject to debate. Proposed ecological mechanisms range from strict local determinism to neutral ecological drift [6]. It is believed that interactions and differences in niche requirements among species play a crucial role in determining the local community structure [5].

Community ecology is a field of study that focuses on the interactions between species in a given area and the effects of these interactions on the environment [7]. An ecological community is a group of species that occupy a specific location and share similar environmental requirements [8]. An ecological community can be represented as a single point in a spatial framework that maps the distribution of species in relation to environmental factors such as temperature, moisture, and topography. It can also be viewed in a temporal framework that tracks changes in population dynamics and biogeographic patterns over time in response to environmental fluctuations [7]. Environmental heterogeneity has a significant impact on the structure of ecological communities [9]. Community ecologists study the composition and structure of biotic assemblages, including patterns of spatial variation and dynamics in response to changing environments [7]. Changes in plant communities along physical environmental gradients at various spatial scales (local or regional) have been utilized to measure and forecast the influence of local or regional environments on plant diversity [10] and ecological relationships.

Similarly, topographic factors exert a greater influence on spatial distribution of vegetation [11]. Variables such as altitude, slope, and other environmental factors significantly impact species distribution and plant community types [12]. Among the three main topographic factors controlling vegetation distribution and patterns in mountainous regions, elevation holds the utmost importance [13, 14]. Elevation, together with aspect and slope, influences the microclimate and, consequently, the large-scale spatial distribution and patterning of vegetation Of these three factors, elevation is the most important [14].

A study conducted by Kutiel and Lavee [15] demonstrated significant differences in species richness, diversity, and vegetation cover between North- and South-facing slopes, as determined by variation in solar radiation intensity. Moreover, other studies in Ethiopia have shown the significant effect of slope aspect on vegetation in mountainous areas [16]. Conversely, disturbances such as logging and grazing further diminish ecosystem productivity, basal area, and biomass, thereby impacting ecosystem function [17]. According to Tekle and Maryo [18], human influences, grazing, and the number of plant species, all exhibited a significant decrease with increasing altitude.

Furthermore, information on tree species composition and diversity is critical not only for understanding the structure of a forest community, but also for planning and implementing conservation strategies for the community [17]. Various studies on plant ecology have been conducted in Ethiopia to examine the distribution of plant community types in relation to environmental factors [12, 19]. However, there are limitations to research on the relationships between environmental factors, plant species distribution, and community types in the Gozamin district of Northwestern Ethiopia. Therefore, studying the composition and structure of the forest community can help conserve and understand the state of plant populations and diversity [20]. The objective of this study is to investigate plant community classification and assess the relationship between plant community types and environmental variables in the study area. we hypothesize that the forest patches in the study area has different plant community types and are affected by various environmental variables.

## Materials and methods

### Description of the study area

**Fig. 1:**
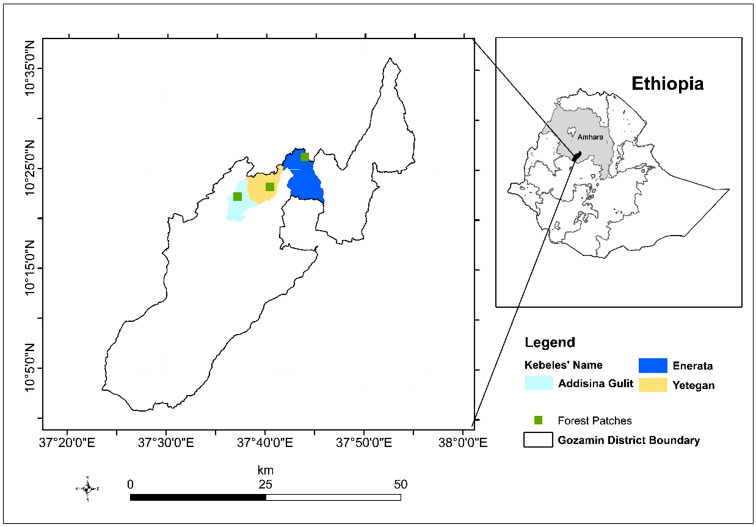
Map of Ethiopia showing the Amhara region, the study district, and the study patches in the Gozamin district in Northwest Ethiopia

The research was conducted in the Forest patches located in Gozamin district of Northwest Ethiopia, situated in the East Gojam zone (Fig. 1). Gozamin district is one of the 20 woredas comprising the East Gojam zone in the Amhara National Regional State. The total area of the district is 121,781 ha, out of which 50,084 ha is arable land, 18,966 ha is grazing land, 22,225 ha is forest (consisting of forest patches), and 30,506 ha is used for other purposes [21].

For this particular study, three forest patches were selected that were relatively undisturbed and had easier transport access. These forest patches are located at elevations ranging from 2300 to 2670 m a.s.l. The mean annual temperature is 20.1^0^ C, with a mean annual rainfall is 1503 mm. The mean minimum and maximum temperatures in the forest patches are recorded as 8.2^0^ and 33.8^0^ C, respectively [22].

The Gozamin district has a total population of 170,690 people, consisting of 85,220 men and 85,470 women [23]. The vegetation in the study area is classified as a dry evergreen Afromontane forest [24]. Subsistence farming is the primary source of income for the local population, with crops such as teff, sorghum, maize, barley, wheat, pulses, and oil crops being the main agricultural products. grown. The homegardens in the area commonly feature fruit trees (e.g. *Prunus persica*), flavoring plants (*Rhamnus prinoides*), stimulant crops (*Coffea arabica* and *Catha edulis*), vegetables (e.g. *Brassica carinata*), medicinal plants (e.g. *Justicia schimperiana* and *Ruta chalepensis*), and ornamental plants [25, 26].

### Reconnaissance survey and sampling technique

A reconnaissance survey was conducted in October 2019, across the forest patches to gain an understanding of the site conditions and identify potential sampling sites. The actual fieldwork took place from October 2020 to April 2021. Three forest patches Melit, Addisinagulit, and Yetegan (Megabzer) were chosen. A systematic sampling technique was used for the collection of vegetation data. Transects were laid along an altitudinal gradient, extending from the ridge top of the mountain where the forest patches were located, to the bottom, where human interference was relatively low. Along elevation gradients, a total of 21 line transects were laid in the forest patches. The number of sampling plots varied per forest patch, based on forest cover, altitudinal differences (gradients), and habitat variability of forest patches (Table 1).

**Table 1:** Size, number of plots, transect length, and altitudinal range of forest patches

	Name of forest patch	Area (Ha)	No of plots	No transect	Altitudinal range (m)	
					Minimum	Maximum
1	Melit	30	33	11	2511	2664
2	Megabzer	15	28	6	2321	2530
3	Addisenagulit	15	15	2	2307	2480

In the forests, the distance between two consecutive plots and the transect was 50 and 150 m above sea level, respectively. A total of 76 sampling plots of each 400 m^2^ (20 m x 20 m) were taken from all forest patches. While for herbaceous species 5 subplots of 2 m x 2 m were laid, four at the corner and one at the center of the main plot. When a study primarily focuses on only woody species composition and diversity, research has largely ignored studies on diversity and not included herbaceous species and rare species, which are important to the understanding of a comprehensive list of plant species diversity in the study area. Consequently, we have incorporated the herbaceous species. To collect herbaceous plant species, the main plot was divided into five 2 × 2 m^2^ smaller subplots, four of which were placed in the corners and one in the center. In our study, the total area of herbaceous species was calculated using the average of the cover values of each species collected from the subplots (4 m^2^).

### Vegetation data collection

The cover and abundance values for all plant species were estimated within each plot. Data tables were created in Microsoft Excel 2016 with three columns and saved in CSV (comma-delimited) format. The first column represents plots, the second column represents species, and the third column represents species abundance. To standardize the percent cover of each species, the values were converted to ordinal scales based on the modified 1–9 Braun-Blanquet scale [27]. The scales for cover-abundance values are 1 ≤ 0.1%, 2 = 0.1 to 1%, 3 = 1 to 2%, 4 = 2 to 5%, 5 = 5 to 10%, 6 = 10 to 25%, 7 = 25 to 50%, 8 = 50 to 75%, and 9 > 75%. The overall cover of herbaceous species in the study was determined by calculating the average individual cover values of species from the subplots (1m^2^). Subsequently, the three-column data containing each species’ cover-abundance value were imported into R statistical software version 4.2.2 [28] and to perform the cluster analysis and other parameters such as Redundancy analysis (RDA)ordination diversity, and evenness. The packages “labdsv” and “vegan” were used to convert the vegetation cover values to the modified Braun Blanquet 1–9 scale the abundance data.

In addition to documenting species within the plots, species discovered outside the plots were also recorded to create a comprehensive floristic list of the study area. After collection, the specimens were coded, pressed, and dried to create voucher specimens. Volumes 1–8 of Flora of Ethiopia and Eritrea were consulted for identification and verification purposes at the National Herbarium (ETH) of Addis Ababa University.

### Environmental data collection

Aspects were coded according to Bekele [29] and Woldu, et al. [30] as N = 0, E = 2, S = 4, W = 2.5, NE = 1, SE = 3, SW = 3.3, and NW = 1.3. Grazing was coded as 0 = nil, 1 = slight, 2 = moderate, and 3 = heavy based [31]. The level of human intervention was estimated based on the presence or absence of stumps, logs, and indicators of fuelwood gathering were quantified as 0 = nil, 1 = low, 2 = moderate, and 3 = heavy [32, 33]. Geographical data (altitude, latitude, and longitude) was recorded using GPS for each plot in the forest patches.

### Data analysis

#### Plant community classification

According to Kent [4] and McCune, et al. [34], cluster analysis assists in grouping a set of observations (plots) based on floristic similarities, i.e., to identify plots that can be classified into the same groups based on species abundance data. In this particular analysis, a data matrix consisting of 76 plots was used, and hierarchical cluster analysis was employed to classify 165 plant species. First, the optimal number of clusters was determined based on Woldu [35]. To identify plant communities in this study, agglomerative hierarchical clustering (AHC) was conducted using the Ward method (minimum-variance clustering) and a similarity ratio (SR). The Multi-Response Permutation (MRPP) procedure was employed to test the clustering of plant communities or groups. The distance matrix used was the same for classification and MRPP The cluster analysis was performed using, R statistical software version 4.2.2 [28], along with the Cluster and Vegan packages [35, 36].

To identify diagnostic species for plant community naming, an indicator species analysis was performed using the indicator value (IndVal) method in the R packages cluster, Vegan, and Labdsv [35, 37]. The indicator value index (IndVal) is based on the cover-abundance and occurrence of a given species within a given set of samples. A species with a significant indicator value of *p* < 0.05 is considered an indicator species of a community in this analysis. The plant community types were then named after two of the dominant species that had a *p* < 0.05 indicator value.

#### Species diversity

Shannon-Wiener diversity index and Shannon’s evenness were calculated to describe the diversity and evenness of plant communities as described by Kent and Coker [38].

$$H=-{\sum }_{i=1}^{n}pi\text{l}\text{n}\left(pi\right)$$

where H = Shannon–Wiener diversity index of the species.

s = number of species, pi = proportion of the species,

ln = the natural logarithm.

Shannon’s evenness index (*J)* was calculated using $J=\frac{H}{H max}$ as well.

where H denotes the Shannon-Wiener Diversity Index and H max = lns, denotes the number of species in the plot.

#### The similarity between communities and forest patches

Ecological resemblance refers to the similarity or dissimilarity between samples in terms of their species composition - two samples with the same species in the same abundances have the highest similarity (and the lowest dissimilarity), and the similarity decreases and the dissimilarity increases as their species composition differs [35]. The sørensen similarity index is among the most commonly used indices. The most commonly used index for comparing plant communities or associations, as well as forest patches and study areas, Sørensen’s similarity index, was used in R statistical program version 4.2.2. was used [28]. The Sørensen’s similarity index, also known as the Sørensen’s similarity coefficient, is a statistic that compares the similarity of two samples, communities, or forest patches. When applied to quantitative data, the formula is abbreviated as:

$${S}_{s}=\frac{2a}{2a+b+c}$$

Where a is the number of species common to both samples, b is the number of species in sample 1, and c is the number of species in sample 2.

#### Ordination

According to McCune and Grace [34], Ordination is a multivariate method that uses ordination diagrams to articulate the relationships between species, plots, and environmental variables in a low-dimensional space.

Preliminary analysis of vegetation data using Detrended correspondence analysis (DCA) can aid in determining the appropriate methods to use in the analysis. If the first axis length is greater than 4, the unimodal method should be used for analysis, and if it is less than 3, the linear model should be used. When the range is between 3 and 4, both types of ordination methods work reasonably well [39]. The length of the first DCA axis in this study was 3.007 indicating the data was homogeneous and giving the chance to choose. Thus, the Redundancy analysis (RDA) ordination method was utilized for this analysis to test the correlations between vegetation and environmental factors. RDA was chosen because it is a method that combines regression and PCA [40]. And also to analyze data on community composition, RDA is a very useful tool [40].

Also, preliminary analysis of the data by Pearson’s correlation coefficient was calculated to find correlations between environmental variables and to remove auto-correlated variables from the ordination analysis using R statistical software version 4.2.2 [28].

A total of Seventy-six sample plots, 165 species, and 5 environmental factors (slope, altitude, aspect, grazing, and human interface) were used in this analysis. The Adonis 2 test was used to investigate the effect of environmental factors on plant community distribution. As a result, RDA ordination was plotted using plant species cover-abundance values and data on significant environmental variables.

## Results

### Floristic composition

In the study area, 168 vascular plant species from 66 families were recorded, out of which 34 species (20.1%) were trees, 52 species (31%), were shrubs, 69 species (40%) were herbs, and 13 species (7.7%) were Lianas. Of the total number of woody species recorded in this study (86 species),52 were shrubs and 34 were trees. Three species namely *Verbascum sinaiticum*, *Scadoxus multiflorus*, and *Cyperus bulbosus* were recorded from outside the plots. Of the total plant species composition, 20(12%) species in 12 families are endemic to Ethiopia and Eritrea. From this, those species under the red list [41] *Dombeya kefaensis, Inula confertiflora*, *Sparmannia macrocarpa*, and *Barleria grandis* are found in the forest patches.

### The dissimilarity between forest patches

The sampled forest patches show less dissimilarity (S < 0.346) that has a high similarity value (S > 0.654) (Table 2).

**Table 2:** The dissimilarity between forest patches

	Addisinagulit	Melit
Melit	0.333	
Yetegan	0.33	0.346

### Plant community types

**Fig. 2:**
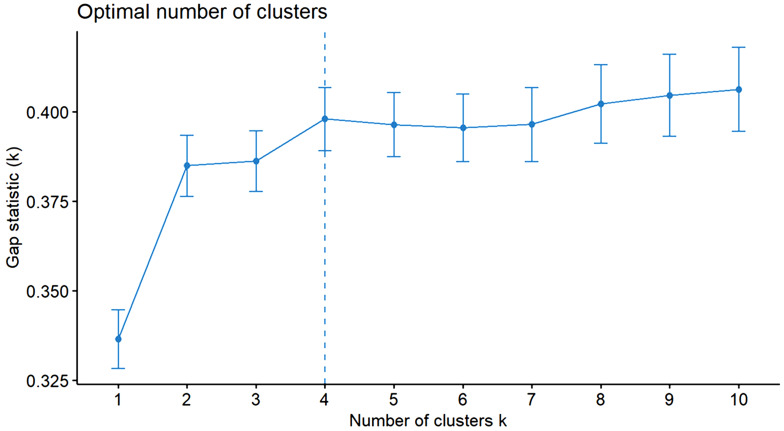
Determining the optimal number of clusters in sampled forest patches

**Fig. 3:**
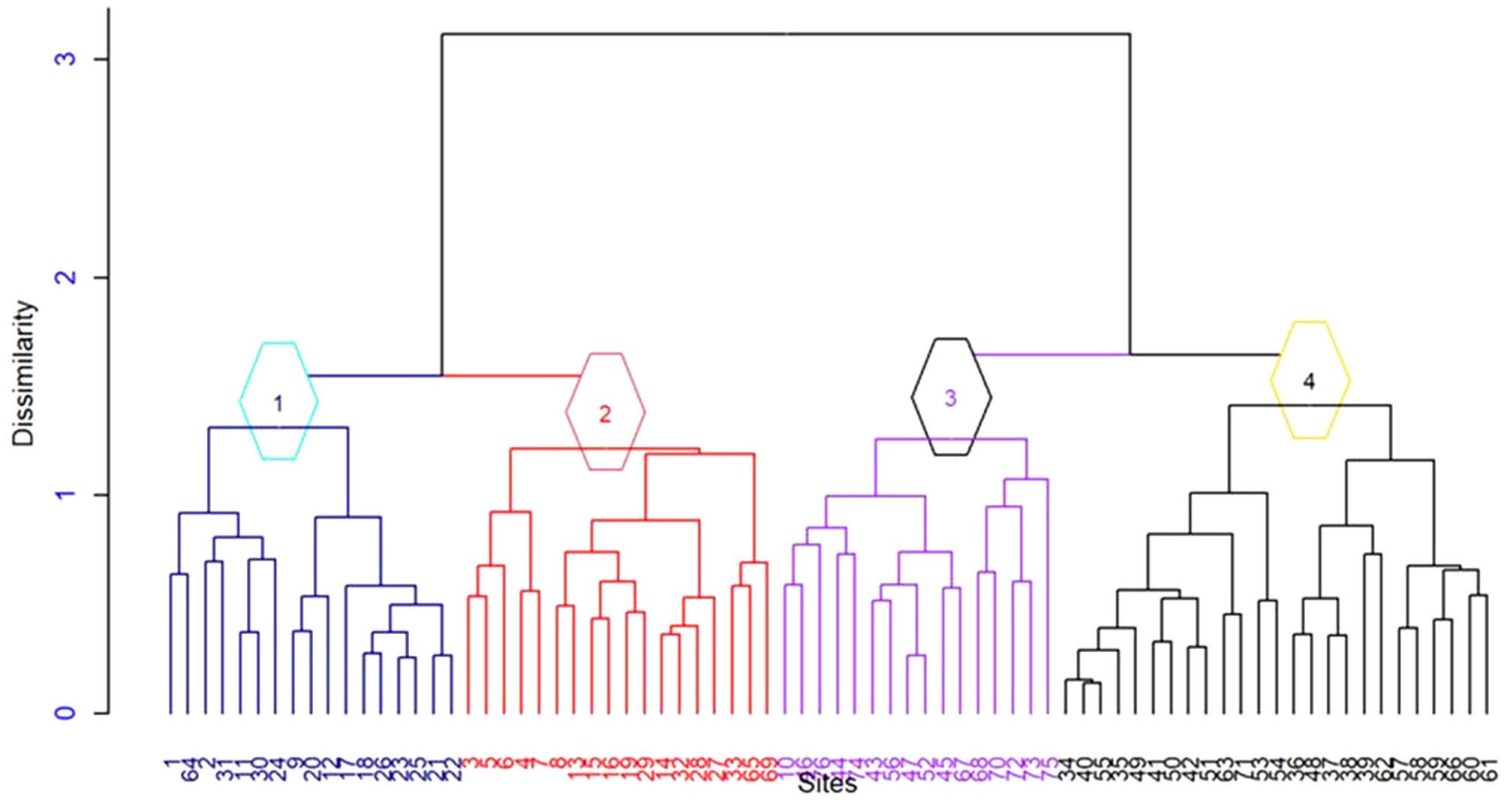
Dendrogram of the vegetation data obtained from hierarchical cluster analysis of the study area (l = Community type 1, 2 = Community type 2, 3 = Community type 3, 4 = Community type 4). Looking into the clusters, I would say that there are only three community types

Four plant community types were identified from the hierarchical cluster analysis in the study area (Figs. 2 and 3). The MRRP test reveals a statistically significant difference in floristic compositions among the four plant communities (R = 0.628, *p* = 0.001). The community is named after one or two dominant indicator tree or shrub species selected by the relative magnitude of their indicator values. In this study, a species is considered an indicator of a group when its indicator value is significantly higher at *p* < 0.05(Table 3). Consequently, the identified communities were *Juniperus procera*- *Olinia rochetiana, Euphorbia abyssinica*- *Dovyalis abyssinica, Albizia schimperiana*-Ficus sur, *Croton macrostachyus -Acacia nilotica*, and *Calpurnia aurea* subsp *Aurea*- *Capparis tomentosa* community type.

**Table 3:** The indicator covers the abundance values of species as well as their significance (*p* < 0.005) in at least two community types in Gozamin forest patches. Values in bold refer to species used to name community types

Indicator species	Indicator value %				
	C1	C2	C3	C4	*P* value
*Juniperus procera* Hochst. ex Endl.	**60**	2	0	0	0.001
*Olinia rochetiana* A. Juss.	**60**	6	0	0	0.001
*Maytenus arbutifolia* (A. Rich.) Wilczek	51	23	0	0	0.001
*Pittosporium viridiflorum* Sims.	47	16	0	0	0.001
*Myrica salicifolia* Hochst. ex A. Rich.	**45**	0	0	0	0.001
*Myrsine africana* L.	**24**	0	0	0	0.003
*Euphorbia abyssinica* Steud. ex A. Rich.	2	**71**	0	0	0.001
*Dombeya torrida* (J. F. Gmel.) P. Bamps	0	**29**	3	0	0.001
*Allophylus abyssinicus* (Hochst.) Radlk.	19	33	2	1	0.003
*Dovyalis abyssinica* (A. Rich.) Warb.	18	40	1	0	0.001
*Albizia schimperiana* Oliv.	0	0	**50**	6	0.001
*Ficus sur* Forssk.	0	0	**19**	0	0.007
*Croton macrostachyus* Del.	0	1	13	**51**	0.001
*Acacia nilotica* (L.) Willd. ex Del.	1	0	0	**35**	0.001
*Acacia abyssinica* Hochst. ex. Benth.	2	1	22	43	0.001
Calpurnia aurea (Ait.) Benth. subsp Aurea	2	7	9	46	0.001
*Osyris quadripartita* Decn.	29	2	0	6	0.006
*Nuxia congesta* R. Br. ex Fresen.	26	9	1	9	0.019
*Rhus vulgaris* Meikle.	26	13	9	1	0.031
*Gnidia glauca* (Fresen.) Gilg.	24	0	0	0	0.004
*Rhamnus staddo* A. Rich.	23	3	0	0	0.007
*Prunus africana* (Hook.f.) Kalkm.	21	22	1	0	0.028
*Bersama abyssinica* Fresen.	21	18	28	1	0.037
*Stephania cyanantha* Welw.ex Hiern.	19	6	0	0	0.031
*Brucea antidysenterica* J. F. Mill.	3	12	11	31	0.018
*Urera hypselodendron* (A. Rich) Wedd.	3	22	10	0	0.037
*Solanecio gigas* (Vatke) C. Jeffrey.	1	4	17	1	0.05
*Albizia schimperiana* Oliv.	0	0	50	6	0.001
*Asparagus africanus* Lam.	0	0	1	27	0.004
*Inula confertiflora* A.	0	0	18	2	0.018
*Hypericum gnidiifolillm* A.Rich.	0	0	15	2	0.029
*Vernonia amygdalina* Del.	0	24	4	2	0.009
*Impatiens rothii* Hook. f.	0	1	12	1	0.039
*Entada abyssinica* Steud. ex A. Rich.	0	0	13	0	0.035
*Dombeya torrida* (J. F. Gmel.) P. Bamps	0	29	3	0	0.001

#### *Juniperus procera- Olinia rochetiana- Myrica salicifolia* community type (C1)

This community type is found in the Melit forest patch, which has an altitudinal range of 2511–2664 m a.s.l. The community contains seven indicator species with significant indicator values: *Juniperus procera*, *Olinia rochetiana*, *Maytenus arbutifolia*, *Pittosporium viridiflorum*, *Myrica salicifolia*, *Apodytes dimidiate*, and *Clausena anisate*. The shrub layer in this community was indicated by *Myrsine Africana, Carissa spinarum*, *Gnidia glauca*, and *Osyris quadripartite*. The climber species common in this community was *Stephania cyanantha*. *Peucedanum winkleri* comprises the herb layer. This community type contains 94 species.

#### *Euphorbia abyssinica- Dombeya torrida* community type (C2)

This community type is found in Melit forest patches, similar to community one, in the elevation range of 2511-2664ma.s.l. This community type is represented by 107 species and has four indicator species: *Euphorbia abyssinica*, *Dombeya torrida, Dovyalis abyssinica*, and *Allophylus abyssinicus*. *Carissa spinarum* was the indicator species in the shrub layer in this community type. The most common climber species in this community type is *Urera hypselodendron*.

#### *Albizia schimperiana- ficus sur* community type (C3)

This community type which has 106 species is found in Adisenagulit and Yetegan forest patches with elevations ranging from 2321 to 2480 m a.s.l. This community’s indicator species included *Albizia schimperiana*, *Ficus sur*, and *Bersama abyssinica*. *Acalypha psilostachya*, *Solanecio gigas*, *Inula confertiflora*, *Vernonia myriantha*, *Entada abyssinica*, and *Hypericum gnidiifoliillm* are indicator shrubs in this community. *Urera hypselodendron* was the most commonly found climber plant species in this community. *Impatiens rothii*, *Justicia heterocarpa*, *Utrica simensis*, and *Aeschynomene abyssinica* were common in the herb layer in this community.

#### *Croton macrostachyus -acacia nilotica* community type (C4)

The dominant community types in Addisenagulit and Yetegan forest patches, which are made up of 95 different species, are located between 2321 and 2480 m a.s.l. *Croton macrostachyus*, *Acacia nilotica*, *Acacia abyssinica*, and *Brucea antidysenterica* were the indicator species in this community type. *Calpurnia aurea* subsp *Aurea, Asparagus africanus, Carissa spinarum*, and *Asparagus africanus* are the shrub species that indicate the shrub layer. *Hypoestes triflora*, *Kalanchoe petitiana*, *Echinops macrochaetus*, and *Peucedanum winkleri* comprise the herb layer.

### Diversity and evenness of plant communities

Shannon’s diversity index revealed that communities 2 and 3 had the greatest species diversity, followed by community 1 and that community 4 had the least. And also, community 3 had the highest evenness value, followed by communities 1 and 2. Communities 4 had the lowest evenness values (Table 4).

**Table 4:** Diversity and evenness of plant communities

Plant community types	Richness	Shannon Diversity(H)	Evenness (J)
Comminty 1	94	3.612	0.795
Comminty 2	107	3.664	0.784
Comminty 3	106	3.716	0.797
Comminty 4	95	3.40	0.754

### The dissimilarity between plant communities

The dissimilarities between plant communities were generally small (S < 0.5). The smallest dissimilarity was between C3 and C4, C2 and C4 as well as C2 and C3 (Table 5). The highest dissimilarity was between C1 and C3. The number of species shared between C1 and communities C2, C3, and C4 was 63, 52, and 59 respectively.

**Table 5:** Sorensen dissimilarity value that shows dissimilarity between community’s

	C1	C2	C3
**C2**	0.363	1	
**C3**	0.48	0.343	1
**C4**	0.407	0.337	0.284

### Correlation among environmental variables

Altitude was negatively correlated with slope, grazing, and human interference (*p* < 0.05) (Table 6).

**Table 6:** Pearson correlation coefficients between environmental variables in Gozamin District, Northwestern Ethiopia

	Slope	Aspect	Grazing	Human interference	Attitude
Slope	1.00				
Aspect	0.14	1.00			
Grazing	0.28**	0.09	1.00		
Human interference	0.37***	0.23*	0.49***	1.00	
Altitude	-0.58***	-0.14	-0.47***	-0.30**	1.00

Significant codes: 0.001 ‘***’ 0.01 ‘**’ 0.05 ‘*’

### Relationship between plant community types and environmental variables

#### Detrended correspondence analysis (DCA)

DCA analysis was performed to determine whether the species were responding linearly or unimodal to environmental gradients. The DCA output of our dataset revealed that the first axis length was 3.007 (Table 7).

**Table 7:** DCA output of vegetation composition in the data set

	DCA1	DCA2	DCA3	DCA4
Eigenvalues	0.459	0.263	0.204	0.193
Decorana values	0.462	0.304	0.209	0.186
Axis lengths	3.007	3.968	3.063	2.474

#### RDA ordination

**Fig. 4:**
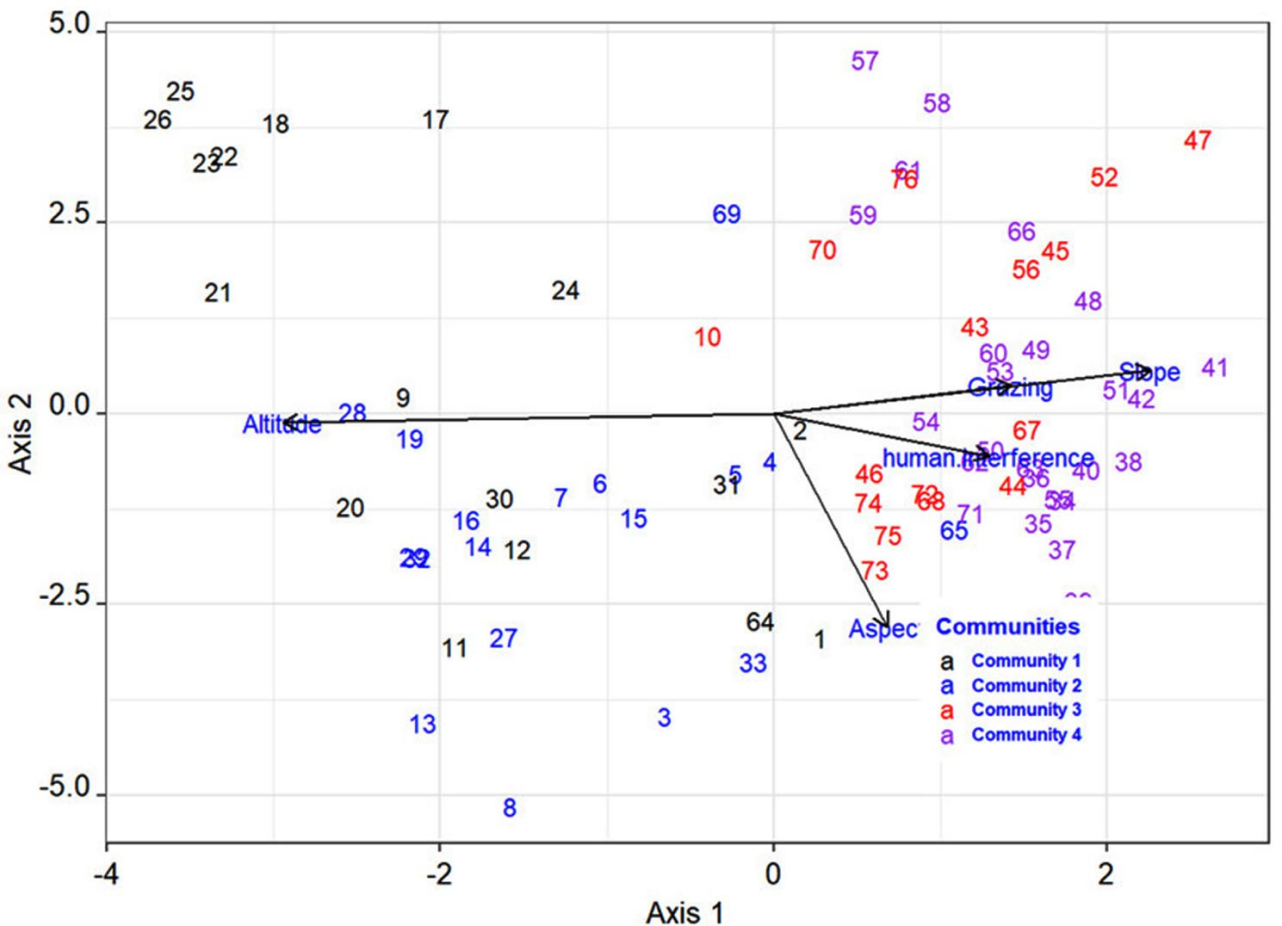
Redundancy analysis (RDA) ordination graph of significant environmental variables (*p* < 0.05) and the plant community in the study area

As shown in Table 8; Fig. 4, of the six environmental variables included in the ordination analysis, (slope, aspect, grazing, and altitude were significantly (*p* < 0.05) correlated with community species composition and distribution. Among significant variables, slope and altitude have high variability. A high sum of squares indicates that there is a lot of variability in the data, whereas a low sum of squares (most of the measurements are close to the mean) indicates that there isn’t much variability (table). According to the RDA diagram, the first axis was primarily correlated with altitude, slope, and grazing, whereas the second axis was primarily correlated with aspect and human interface (Fig. 4; Table 9).

**Table 8:** Result of function adonis2 test of environmental variables (significant environmental variables and their p-value) in the study area

Environmental variables	Df	Sum of sqs	R2	F	Pr(> F)
Altitude	1	1.6043	0.07875	7.3534	0.001***
Slope	1	2.0724	0.10173	9.4992	0.001***
Aspect	1	0.4754	0.02333	2.1789	0.027*
Grazing	1	0.7490	0.03677	3.4332	0.003 ***
Human interference	1	0.1992	0.00978	0.9130	0.454
Residual	70	15.2720	0.74964		
Total	75	20.3723	1.00000		

**Table 9:** Biplot scores for constraining variables and their correlation with the RDA axis, eigenvalues, and proportion of variance explained

	RDA1	RDA2	RDA3	RDA4	RDA5
Altitude	-0.97	-0.036	-0.024	-0.035	0.2371
Slope	0.75	0.185	-0.236	0.042	0.5949
Aspect	0.22	-0.919	0.001	0.221	0.234
Grazing	0.46	0.123	0.808	0.3012	0.149
Human interference	0.42	-0.181	0.533	-0.581	0.406
Eigenvalues	48.918	6.225	4.597	2.68	1.859
Proportion Explained	0.761	0.0968	0.0715	0.0417	0.0289
Cumulative Proportion	0.761	0.8578	0.92937	0.9711	1.000

Significant codes: 0.001‘***’ 0.01 ‘**’ 0.05 ‘*’

## Discussion

### Diversity of forest patches

We found a relatively high diversity of plant species in the forest patches of Gozamin district. The forest patches are DAF forest type. According to Friis, et al. [42], the dry evergreen Afromontane Forest and grassland complex (DAF) is the second richest vegetation type after Acacia-Commiphora woodland and bushland (ACB). The sampled forest patches exhibit a high level of similarity S > 0.6 (Table  1) sharing 60% of the species. The two patches found in Addisinagulit and Yetegan are negatively impacted by human settlement and farming. Additionally, the sampled forest patches show high similarity, sharing 77% of the species.

The current study reveals a lower number of woody species compared to similar studies conducted in the Afromontane Forest of Northwestern Ethiopia, specifically in the Dega Damot district [43], in Baso Liben, and Debre Elias Districts [44], in Sesa Mariam Monastery [45]. However, the species richness in our study is higher compared to research conducted in dry Afromontane forests, such as the Kuandisha Afromontane forest fragment [46]. The differences in species richness among these forests could be attributed to geographical location, altitude, anthropogenic impact, rainfall, and other climatic, physiographic, and edaphic factors [47].

The findings from the study area revealed a relatively high endemicity (12%). This is due to endemic plant species found in Ethiopia’s montane forests, which make up 10.7% of the country’s total species, account for endemic plant species [3]. Gebrehiwot et al. [11] discovered that 21% of the floristics were endemic to north Ethiopia. Northwest Ethiopia has 10.6% endemic species, according to Birhanu and Bekele [12]. The two dominant families in the study area are Asteraceae and Fabaceae. According to other research, Asteraceae and Fabaceae predominate in dry evergreen Afromontane forests [12, 45, 48]. Asteraceae and Fabaceae are two of the most widely distributed vascular plant families, ranking third and first in the Ethiopian flora, respectively [3].

The outcrossing behavior of Asteraceae inflorescences and the diverse pollination mechanisms likely contributed to the family’s cosmopolitan [49]. And also these are the top species-rich families in terms of global distribution [50, 51].

### Plant community types

The research area’s forest patches comprised four distinct plant community types, which were identified using hierarchical cluster analysis and ordination methodologies. The identified plant communities are diverse and species-rich. Even if the forest patches are found in the altitude range of 2300 and 2670 m a.s.l., the patches have a mountainous and slopy type and have 4 community types. Although the similarity of the forest patches has high similarity (S > 0.5) and less dissimilarity (S < 0.5), Due to the community’s location, these have relatively similar environmental factors. The MRRP test shows the four community types have significant differences (R = 0.628, P = 0.001). However, the difference could be directly related to environmental factors that cause plant communities to have distinct or distinguishing species [52]. This is also supported by the findings of Bhattarai, et al. [53], Lovett [54], and Tekle and Maryo [18] that showed in mountainous ecosystems, there is a rapid shift in altitudinal gradients, temperature, rainfall, and drainage. As a result, the diversity of plants varies within a short distance. The area’s vegetation is classified as a dry evergreen Afromontane Forest (DAF) [24] which is dominated by *Juniperus procera*, *Olinia rochetiana*, *Myrica salicifolia*, *Euphorbia abyssinica*, *Dombeya torrida*, *Albizia schimperiana*, *Ficus sur*, *Croton macrostachyus*, *and Acacia nilotica*.

Community1 (*Juniperus procera*- *Olinia rochetiana*- *Myrica salicifolia*) and community 2 (*Euphorbia abyssinica*- *Dombeya torrida*) are strongly associated with higher elevation and are found in the Melit forest patch, which is dominated by large woody plants.

Community 2 (*Euphorbia abyssinica*- *Dombeya torrida* community type) and Community 3(*Albizia schimperiana*- *Ficus sur)* had the greatest species richness and diversity. Community 1(*Juniperus procera- Olinia rochetiana- Myrica salicifolia*) and Community 4 (*Croton macrostachyus* -*Acacia nilotica*), on the other hand, had the less species richness and diversity of the remaining community types. The high species diversity and richness of community 2 could be due to the altitudinal range (2511–2664 m.a.s.l) which is found in the Melit forest patch with relatively low disturbance. Also, species at higher altitudes have higher diversity due to low disturbance [55]. Furthermore, grazing and human interaction are less effective in the community due to most of the plants have no significant effect on economic value. Community 4 is the most disturbed community type that is found in Addisinagulit and Yetegan forest patches (Fig. 4) by human interference like cutting of plants for making different materials, fuel, and grazing. And also, differences in terms of altitude, slope, aspect, and other environmental factors affect communities (Table 7). The present finding was slightly consistent with the finding of Mokarram and Sathyamoorthy [13], who showed that the primary determinant of vegetation growth is topography. Similarly, the vegetation study in Sesa Mariam Monastery, Northwestern Ethiopia conducted by Meshesha and Tsegay [45], showed the grazing effect on vegetation composition.

### Environmental factors and plant community relationship

The patterns of floristic grouping within the studied forest patches were consistent across multivariate analyses (both RDA ordination and cluster analysis) (Figs. 3 and 4). The analysis of vegetation data using cluster analysis and ordination techniques can provide more detailed and comprehensive information on vegetation patterns and plant species responses to major environmental factors [56]. The RDA analysis of environmental, edaphic, and topographic data from the forest suggested that variation in topographical features influenced the formation of four plant communities. As a result, the heterogeneous habitat required for the formation of different plant communities may have been created. The four communities were shown by the length of the first axis by the DCA axis is an indication of the influence of environmental factors influencing species composition and high species diversity in four plant communities. In this study, the distribution of plant communities represents the combined influence of altitude, slope, grazing, aspect, and human interface elements, which supports the hypothesis.

Among these, factors altitude was the most important environmental variable in explaining variations in plant species distribution and patterns of plant community formation, according to multivariate analyses (both RDA Ordination and cluster analysis) because it influences atmospheric pressure, moisture, and temperature, all of which govern plant growth and distribution.

Environmental variables that were highly correlated with axis one was largely responsible for explaining RDA with a higher axis score. The variable associated with axis one with the highest score was altitude. As a result, altitude was the most important variable in weighting axis one and explaining or interpreting the axis. Factors grazing, slope, and human interference have reverse effect with altitude (Fig. 4) this agrees with Tekle and Maryo [18] that show that human influences, such as grazing, decreased significantly as altitude increased. Similar research findings in Ethiopian Afromontane forests confirmed the importance of altitude as a major determinant of vegetation distribution along altitudinal gradients [12, 19, 57]. According to Lenoir, et al. [58], changes in species distribution are associated with altitudinal range. which, in turn, determines the patterns of vegetation distribution.

The aspect was also the most important constraining variable in weighing axis two in the ordination. That shows have a great effect on Gozamin forest patch distribution. This was also supported by a study in the mountain area of Iran by Mokarram and Sathyamoorthy [13] that showed elevation and aspect affect vegetation. Similarly, the slope had a significant effect on axis one. This is supported by Busing and White [14]. Furthermore, grazing had a significant impact on plant communities in Gozamin forest patches. Gebeyehu, et al. [59] also show the grazing effect on plant composition in the Awi zone in Northwest Ethiopia. Dibaba andSoromessa [19], and Bussmann [60] assured that disturbance affects plant community distribution by impeding natural regeneration and seedling establishment in tropical forests. And in general, altitude, aspect, slope, and grazing have a significant effect (Table 7) on the study area’s forest patch composition and community formation and this is also supported by other studies [11–14].

A positive and significant correlation was observed between human interference, slope, aspect, and grazing but a negative correlation with altitude. The negative correlation between altitude and grazing and human interference was explained by the fact that the disturbance factors decreased at high altitudes. Human interference and grazing effect have a positive correlation in the lower altitude.

The eigenvalue of the RDA’s first axis (48.9) was significantly greater than the eigenvalues of the RDA’s second axis (6.2). (Table 7). This demonstrates that the first axis has the greatest influence on the variation, and thus it is the most important environmental variable, accounting for approximately 76% of the total variation. The role of altitude gradients in shaping the distribution of vegetation in East African mountains had previously been reported by [11, 61, 62]. The study area’s elevation has a highly significant impact on the development of plant community formation, and grazing and human disturbance have a negative correlation with elevation. A conservation strategy taking these factors into account can help prevent forest disturbances.

## Conclusion

In this study a total of 168 vascular plant species from 66 families were discovered, of which 12% were endemic to the area. Among these, 7% were listed in the red list of plants in Ethiopia and Eritrea. This study identified four plant communities. Among all the investigated environmental factors, elevation, slope, aspect, and grazing were found to significantly explain variation in species composition and community formation in the study area. Elevation was found to be the most important environmental factor influencing species distribution and community formation.

The overall findings emphasize the importance of considering the disturbance effect of floristic composition in conservation plans. Our results suggest that conservation schemes need to consider the impact of grazing on floristic composition in the management of forest patches to conserve plant diversity, including species listed in the red list of Ethiopia and Eritrea. Altogether, Our results suggest that conservation schemes should take into account the impact of grazing and human interference on floristic composition for managing the plant diversity of forest patches, which includes species listed in the red list of Ethiopia and Eritrea. Additionally, more research into environmental factors that affect forest patches, such as soil taste analysis and their effect on forest distribution, as well as more research into removing disturbance factors to conserve forest patches, is needed.

## Data Availability

All data analyzed during this study are included in this article.

## References

[1] Friis I, Thulin M, Adsersen H, Bürger A-M (2005). Patterns of plant diversity and endemism in the Horn of Africa. Biol Skr.

[2] Dragan M, Feoli E, Fernetti M, Zerihun W (2003). Application of a spatial decision support system (SDSS) to reduce soil erosion in northern Ethiopia. Environ Model Softw.

[3] Demissew S, Friis I, Weber O (2021). Diversity and endemism of the flora of Ethiopia and Eritrea: state of knowledge and future perspectives. Rend Lincei Scienze Fis E Naturali.

[4] Kent M. Vegetation description and data analysis: a practical approach. John Wiley & Sons; 2011.

[5] Svenning J-C, Kinner DA, Stallard RF, Engelbrecht BM, Wright SJ (2004). Ecological determinism in plant community structure across a tropical forest landscape. Ecology.

[6] Wright JS (2002). Plant diversity in tropical forests: a review of mechanisms of species coexistence. Oecologia.

[7] Jackson ST, Blois JL (2015). Community ecology in a changing environment: perspectives from the Quaternary. Proc Natl Acad Sci.

[8] Ricklefs RE (2008). Disintegration of the ecological community: American Society of naturalists Sewall Wright award winner address. Am Nat.

[9] Vivian-Smith G. Microtopographic heterogeneity and floristic diversity in experimental wetland communities. J Ecol. 1997:71–82.

[10] Willis KJ, Whittaker RJ (2002). Species diversity–scale matters. Science.

[11] Gebrehiwot K, Woldu Z, Fekadu M, Teferi E, Desalegn T, Demissew S (2020). Classification and ordination of plant communities in Abune Yosef mountain range, Ethiopia. Acta Ecol Sin.

[12] Birhanu L, Bekele T, Tesfaw B, Demissew S (2021). Relationships between topographic factors, soil and plant communities in dry afromontane forest patches of Northwestern Ethiopia. PLoS ONE.

[13] Mokarram M, Sathyamoorthy D (2015). Modeling the relationship between elevation, aspect, and spatial distribution of vegetation in the Darab Mountain, Iran using remote sensing data. Model Earth Syst Environ.

[14] Busing R, White P, MacKenzie M (1993). Gradient analysis of old spruce–fir forests of the Great Smoky Mountains circa 1935. Can J Bot.

[15] Kutiel P, Lavee H (1999). Effect of slope aspect on soil and vegetation properties along an aridity transect. Isr J Plant Sci.

[16] Gebrelibanos T, Assen M (2014). Effects of slope aspect and vegetation types on selected soil properties in a dryland Hirmi watershed and adjacent agro-ecosystem, northern highlands of Ethiopia. Afr J Ecol.

[17] Malik ZA, Bhatt A. Phytosociological analysis of woody species in Kedarnath Wildlife Sanctuary and its adjoining areas in Western Himalaya, India. JFES.2015, 31(3):149–163.

[18] Tekle T, Maryo M (2022). Ecological Assessment of Woody Plant Diversity and the Associated threats in Afromontane Forest of Ambericho, Southern Ethiopia. J Landsc Ecol.

[19] Dibaba A, Soromessa T, Warkineh B (2022). Plant community analysis along environmental gradients in moist afromontane forest of Gerba Dima, South-western Ethiopia. BMC Ecol Evol.

[20] Benchimol M, Mariano-Neto E, Faria D, Rocha-Santos L, de Souza Pessoa M, Gomes FS, Talora DC, Cazetta E (2017). Translating plant community responses to habitat loss into conservation practices: forest cover matters. Biol Conserv.

[21] GAO. Gozamin agricultural office report. In.; 2021.

[22] National Aeronautics and Space. Administration’s power access data viewer. 〈https://power.larc.nasa.gov/data-access-viewer/〉 (accessed 9 March 2023).

[23] CSA: Ethiopian Population Pyramid. Central Statistical Agency, Addis Ababa. In.; 2007.

[24] Friis I, Demissew S, Van Breugel P. Atlas of the potential vegetation of Ethiopia. Volume 307. Det Kongelige Danske Videnskabernes Selskab Copenhagen; 2010.

[25] Yinebeb M, Lulekal E, Bekele T, Lemessa D (2022). Homegardens plant species richness and their use types have positive associations across agricultural landscapes of Northwest Ethiopia. Global Ecol Conserv.

[26] Yinebeb M, Lulekal E, Bekele T (2022). Composition of homegarden plants and cultural use in an indigenous community in Northwest Ethiopia. J Ethnobiol Ethnomed.

[27] Van der Maarel E (1979). Transformation of cover-abundance values in phytosociology and its effects on community similarity. Vegetatio.

[28] R Core Team. R: A Language and Environment for Statistical Computing v4. 2.2 (Version 4.2. 2). 2021.

[29] Bekele T (1994). Phytosociology and ecology of a humid afromontane forest on the central plateau of Ethiopia. J Veg Sci.

[30] Woldu Z, Feoli E, Nigatu L. Partitioning an elevation gradient of vegetation from southeastern Ethiopia by probabilistic methods. Numer Syntaxonomy 1989:189–98.

[31] Zerihun Woldu, Backéus I (1991). The shrubland vegetation in western Shewa, Ethiopia and its possible recovery. J Veg Sci.

[32] Yeshitela K, Bekele T (2002). Plant community analysis and ecology of afromontane and transitional rainforest vegetation of southwestern Ethiopia. SINET: Ethiop j sci.

[33] Woldemichael LK, Bekele T, Nemomissa S (2010). Vegetation composition in Hugumbirda-Gratkhassu national forest priority area. South Tigray MEJS.

[34] McCune B, Grace JB, Urban DL. Analysis of ecological communities. Volume 28. MjM software design Gleneden Beach, OR; 2002.

[35] Woldu Z (2017). Comprehensive analysis of vegetation and ecological data-basic, concepts, methods. Addis Ababa University.

[36] Oksanen J, Blanchet FG, Kindt R, Legendre P, Minchin P, O’hara R, Simpson G, Solymos P, Stevens MHH, Wagner H (2013). Community ecology package. R Package Version.

[37] De Cáceres M (2013). How to use the indicspecies package (ver. 1.7. 1).

[38] Kent M, Coker P. The description of vegetation in the field. Vegetation description and analysis: a practical approach 1992.

[39] Šmilauer P, Lepš J. Multivariate analysis of ecological data using CANOCO 5. Cambridge university press; 2014.

[40] Borcard D, Gillet F, Legendre P, Borcard D, Gillet F, Legendre P. Canonical ordination. Numer Ecol R 2018:203–97.

[41] Jose Luis V, Ensermu K, Sebsebe D. The Red List of Endemic Trees & Shrubs of Ethiopia and Eritrea: Fauna and Flora International, Global Trees Campaign, IUCN. ISBN: 1 903703 19 0; 2005.

[42] Friis I, Sebsebe D, van Paulo B. Atlas of the potential vegetation of Ethiopia, Addis Ababa. In.: Addis Ababa University Press & Shama Books; 2011.

[43] Birhanu L, Bekele T, Tesfaw B, Demissew S (2022). Soil seed bank composition and aboveground vegetation in dry afromontane forest patches of Northwestern Ethiopia. Trees Forests and People.

[44] Amsalu B. An Ethno botanical study of traditional medicinal plants used in guna begimder woreda, South Gonder zone of Amhara region, Ethiopia. 2020.

[45] Meshesha BW, Tsegay BA, Telake BB (2015). Survey on the composition of perennial vegetation in Sesa Mariam Monastery, Northwestern Ethiopia. BMC Res Notes.

[46] Berhanu A, Demissew S, Woldu Z, Didita M (2017). Woody species composition and structure of Kuandisha afromontane forest fragment in northwestern Ethiopia. J for Res.

[47] Brockway DG (1998). Forest plant diversity at local and landscape scales in the Cascade Mountains of southwestern Washington. For Ecol Manag.

[48] Nigussie A. Vascular Plant Diversity and Ethnobotany of Medicinal and Wild Edible Plants in Baso Liben and Debre Elias Districts, East Gojjam Zone of Amhara Region, Northwestern Ethiopia. Addis Ababa University; 2020.

[49] Cuffia C, Cerino MC, Tomas PA, Exner EL (2022). Winter flowers for bees: reproductive biology of Trixis praestans (Asteraceae). Plant Syst Evol.

[50] Tadesse M (2004). Flora of Ethiopia and Eritrea, Part 2: Asteraceae (Compositae). In.: Addis Ababa.

[51] Judd WS, Campbell CS, Kellogg EA, Stevens PF, Donoghue MJ (1999). Plant systematics: a phylogenetic approach. Ecol Mediterr.

[52] Rahman IU, Hart RE, Ijaz F, Afzal A, Iqbal Z, Calixto ES, Abd Allah EF, Alqarawi AA, Hashem A, Al-Arjani A-BF (2022). Environmental variables drive plant species composition and distribution in the moist temperate forests of Northwestern Himalaya. Pakistan Plos One.

[53] Bhattarai P, Bhatta KP, Chhetri R, Chaudhary RP (2014). Vascular plant species richness along an elevation gradient of the Karnali River valley, Nepal Himalaya. Int j Plant Animal env sci.

[54] Lovett J. Altitudinal variation in large tree community associations on the West Usambara Mountains. Research for Conservation of Tanzania Catchment Forests Uppsala: Uppsala Universitet 1990:48–53.

[55] Chemeda BA, Wakjira FS, Hizikias EB (2022). Tree diversity and biomass carbon stock analysis along altitudinal gradients in coffee-based agroforestry system of Western Ethiopia. Cogent Food Agric.

[56] ter Braak CJF. Ordination. Data analysis in community and landscape ecology. Cambridge University Press; 1995. pp. 91–274.

[57] Lulekal E, Kelbessa E, Bekele T, Yineger H (2008). Plant species composition and structure of the Mana Angetu moist montane forest, south-eastern Ethiopia. J East Afr Nat Hist.

[58] Lenoir J, Gégout J-C, Marquet PA, de Ruffray P, Brisse H. A significant upward shift in plant species optimum elevation during the 20th century. science 2008, 320(5884):1768–1771. 10.1126/science.1156831 18583610

[59] Gebeyehu G, Soromessa T, Bekele T, Teketay D (2019). Species composition, stand structure, and regeneration status of tree species in dry afromontane forests of Awi Zone, northwestern Ethiopia. Ecosyst Health Sustain.

[60] Bussmann RW. Succession and regeneration patterns of east African mountain forests. A review. Syst Geogr Plants. 2001:959–74.

[61] Eshetu EY, Hailu TA (2020). Carbon sequestration and elevational gradient: the case of Yegof mountain natural vegetation in North East, Ethiopia, implications for sustainable management. Cogent Food Agric.

[62] Gebrehiwot K, Demissew S, Woldu Z, Fekadu M, Desalegn T, Teferi E (2019). Elevational changes in vascular plants richness, diversity, and distribution pattern in Abune Yosef mountain range, Northern Ethiopia. Plant Divers.

